# The Effects of Subliminal Goal Priming on Emotional Response Inhibition in Cases of Major Depression

**DOI:** 10.3389/fpsyg.2020.542454

**Published:** 2020-12-22

**Authors:** Man Zhang, Suhong Wang, Jing Zhang, Can Jiao, Yuqi Chen, Ni Chen, Yijia Zhao, Yonger Wang, Shufang Zhang

**Affiliations:** ^1^Department of Psychology, Renmin University of China, Beijing, China; ^2^Department of Clinical Psychology, The Third Affiliated Hospital of Soochow University, Changzhou, China; ^3^School of Psychology, Shenzhen University, Shenzhen, China; ^4^School of Continuing Education, Renmin University of China, Beijing, China; ^5^Research Center for Psychological and Health Sciences, China University of Geosciences, Wuhan, China; ^6^Faculty of Humanities and Social Sciences, City University of Macau, Macau, China; ^7^Wuhan Mental Health Center, Tongji Medical College, Huazhong University of Science and Technology, Wuhan, China

**Keywords:** automatic emotion regulation, subliminal priming, Go/No-Go, response inhibition, depression

## Abstract

Previous studies have provided evidence that automatic emotion regulation (AER), which is primed by control goals, can change emotion trajectory unconsciously. However, the cognitive mechanism and associated changes in depression remain unclear. The current study aimed to examine whether subliminal goal priming could change the emotional response inhibition among patients with major depressive disorder (MDD) and their healthy controls. A group of patients with depression and a healthy control group were both primed subliminally by playing control goal related or neutral words for 20 ms each; afterward, they judged the gender of happy or angry faces in an emotional Go/No-Go task. A group of depressed patients and a healthy control group both were both primed subliminally with control goal-related words (20 ms) or neutral words (20 ms), and they judged the gender of happy or angry faces in an emotional Go/No-Go task. Among patients with depression, there were fewer false alarms of the No-Go response to emotional stimulus after priming with control goal rather than neutral words. Meanwhile, patients with MDD in the subliminal regulation goal priming condition reacted faster to happy rather than angry faces; no significant difference was found in the subliminal neutral priming condition. These findings suggest the malleability of inhibitory control in depression using subliminal priming goals.

## Introduction

Deficits in emotional regulation have been reported to be related to mood disorders, such as depression and anxiety (Rive et al., 2013). Compared with deliberate emotion regulation, which is guided by explicit intention, automatic emotion regulation (AER) modulates human emotions unconsciously and changes emotion trajectory unintentionally (Mauss et al., 2006; Braunstein et al., 2017). Relevant studies have reported a significant relationship between AER and depression vulnerability (McFarland and Buehler, 1998; Koole and Jostmann, 2004; Ehring et al., 2010). Furthermore, experimental studies revealed that depressed persons showed deficient unconscious regulation that produced positive thoughts in response to acute exclusion (DeWall et al., 2011). Zhang and Lu (2012) also found that depressive symptoms among adolescents were negatively correlated with No-Go P3 amplitudes elicited by unconsciously regulating negative emotions in an implicit emotional Go/No-Go task. Studies have evidenced the importance of AER on affective feelings and cognitive processing of emotion in depression, either on correlation or individual difference levels (Ehring et al., 2010; DeWall et al., 2011); however, it remains unclear how AER affects emotion processing in depression. The present study manipulates AER to examine its effect on emotional processing in major depressive disorder (MDD) patients.

In the automotive model, when goals or norms referring to regulating emotions are formed in one’s mind, they can be activated without one’s awareness and influence a related response (Gollwitzer and Bargh, 2005; Bargh and Williams, 2007; Bargh et al., 2012). In many studies, subliminal goal priming has been adopted to promote goal-related behavioral responses on the unconscious level, without substantial cognitive effort from participants (Bargh et al., 2001; Légal et al., 2012; Blakemore et al., 2017). For example, Blakemore et al. (2017) found that both subliminal priming of the goal of tense (frail/tense/abstract) and emotional context modulated unconscious goal activation when participants conducted motor force exertion. Another study found that subliminally priming individuals to be trusting leads to a better evaluation of persuasive messages, increases behavioral intentions in accordance with the message, and positively influences the assessment of the source (Légal et al., 2012). Furthermore, recent studies have found that subliminal priming with the goal of controlling, rather than expressing, emotions may reduce the skin conductance levels of participants with emotion control tendencies (Zhang et al., 2017). Given all the evidence above, subliminal goal priming would be adopted in the present study to manipulate AER.

Literature has revealed that depression patients exhibited deficient response inhibition to negative stimuli. For example, Everaert et al. (2017) found that deficient inhibitory control toward angry faces was related to negative attention bias, which in turn predicted a congruent bias in interpretation and, subsequently, depressive symptoms. Individuals who exhibited high depressive rumination also showed impairments in inhibition of angry faces (De Lissnyder et al., 2010). Furthermore, Schlosser et al. (2013) found that MDD patients showed higher false alarm rates of No-Go trials than healthy controls in a Go/No-Go task with fearful, happy, and neutral stimuli. However, much research has also found no difference in the error rates of No-Go responses between patients with MDD and healthy individuals. For example, Grunewald et al. (2015) found both MDD patients and healthy controls showed lower error rates in happy rather than sad or neutral No-Go trials. Colich et al. (2015) found no group difference in the error rate for emotional No-Go trials behaviorally but revealed that only depressed adolescents showed less activation in the dorsolateral prefrontal cortex for inhibiting No-Go targets that followed a sad rather than happy face.

Similarly, for the reaction time in Go trials, mixed results were found for group difference. MDD patients were found to react faster to negative emotional stimuli than healthy groups (Ladouceur et al., 2006). Han et al. (2012) used the emotional Go/No-Go paradigm and found higher depression scores were related to shorter reaction times to negative emotional faces (angry, fearful, and sad faces) for the Go responses. Conversely, literature has also reported no group difference in the reaction time of depressed and healthy controls in terms of Go response to negative stimuli (Hummer et al., 2013). For example, Grunewald et al. (2015) found no difference between adolescences with MDD and healthy controls in response to emotional (fearful, happy, or sad) Go stimuli. Similarly, Colich et al. (2015) found that adolescents with MDD showed no difference in reaction time during happy or sad Go trials than healthy controls. Finally, Schlosser et al. (2013) reported longer reaction times to happy or fearful Go stimuli in MDD patients than healthy controls.

This study aimed to examine whether subliminal priming of control goals would modulate emotional response inhibition among patients diagnosed with MDD and their healthy controls. We used subliminal priming and an emotional Go/No-Go paradigm in the study. During each trial, participants would first be subliminally primed with a control or neutral goal and then classify the gender of angry or happy faces using Go or No-Go responses. We hypothesized that compared with neutral priming, subliminal priming of control goals would lead to delayed reactions to angry faces in the Go trials and enhanced accuracy toward angry faces in No-Go trials, especially for healthy controls. It was also expected that, for angry faces, MDD patients would show shorter reaction times in Go trials and lower accuracy in No-Go trials under the neutral priming condition, because of their difficulties in AER and emotional response inhibition (Kaiser et al., 2003; Ehring et al., 2010).

## Materials and Methods

### Participants

The sample size was measured using G^∗^Power 3.1. At least 54 participates were required (effect size = 0.25; power = 0.95). For the experimental group, 31 patients (four males; age: mean = 23.19 ± 5.31 years, range = 17–36 years) with a *Diagnostic and Statistical Manual of Mental Disorders, Fifth Edition* (*DSM-5*) diagnosis of MDD and no comorbid *DSM-5* pathologies were recruited from hospitals. Another 28 participants (five males; age: mean = 24.25 ± 4.93 years, range = 17–39 years) were subsequently recruited for the healthy control group.

Inclusion criteria for the MDD group consisted of the following: MDD diagnosed by a psychiatrist based on a structured interview in accordance with the *DSM-5*; no antidepressant medication taken 14 days before enrolment; dosage duration not more than 2 years; aged 17–50 years old; having received more than 9 years of education, with normal intelligence, and no Chinese dyslexia; right-handed; and normal or corrected vision. All participants were recruited from the same clinic and had to adhere to identical criteria. The exclusion criteria were as follows: history of organic brain disease or traumatic brain injury, severe physical illness, other Axis I mental disorders in the past or present, borderline personality disorder, history of electroconvulsive therapy, alcohol dependence/abuse in the past 6 months, and intellectual disability.

For every MDD participant tested, control participants—with no history of depression and with normal or corrected vision—were identified and recruited and were matched by age, gender, and education. The two groups did not differ in age, gender, or education. Screening criteria of the control group included the absence of mood disorders and less than 10 points in the Beck Depression Inventory-II (Chinese version; Wang et al., 2011).

### Materials

#### Priming Words

Participants were primed subliminally using two categories of words: regulation-related and neutral words. The regulation-related words were selected according to work by Mauss et al. (2007). These words were translated by a teacher of English. Some changes were made according to a return translation. Another 30 undergraduate students rated the belongings of each regulation-related and neutral word. The instruction was presented as follows: “You will see some Chinese verbs. Please rate to what extent the word belongs to the category of emotion control, with 1 = very irrelevant, 2 = less relevant, 3 = somewhat relevant, 4 = more relevant, and 5 = very relevant.” Sixteen verbs each for both the regulation [e.g., relieved (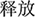), subside (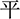

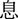
), calm (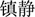)] and neutral [e.g., wave (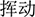), stand (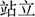), touch (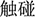)] were selected by more than 3.9 and 1.2 points, respectively. An independent-sample *t*-test showed no significant difference in the word frequency of the two sets of priming words (*p* > 0.05).

#### Face Images

Forty angry facial pictures (20 males), 40 happy facial pictures (20 males), and five practice pictures were selected from the Chinese Facial Affective Picture System, which provided arousal and valance scores for each picture (Gong et al., 2011). Each picture (12 × 10 cm) was presented three times in a pseudorandom order to ensure the same faces did not follow each other. There were no differences in arousal between happy and angry faces [5.87 ± 0.31 vs. 5.71 ± 0.29; *t*(39) = 0.965, *p* > 0.05].

### Procedure

In this study, a three-way mixed design of Go/No-Go tasks with group (between-subject, MDD vs. healthy) × priming (within-subject, subliminal control goal vs. neutral goal) × emotion (within-subject, angry vs. happy) was used.

**FIGURE 1:**
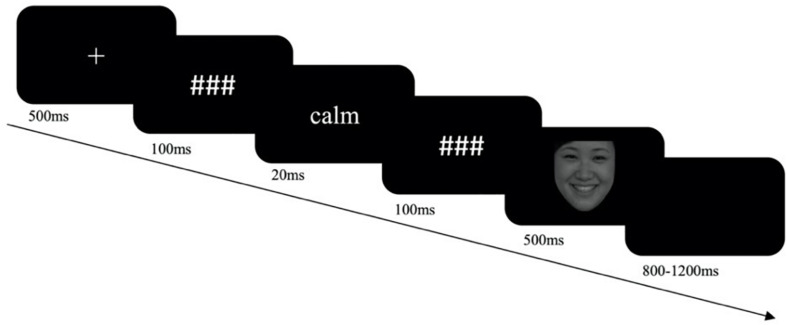
The procedure of a single trial of the experiment. The facial picture in the procedure is from the Chinese Facial Affective Picture System (Gong et al., 2011).

The experiment consisted of two sessions with 520 trials in total, with one session each for the subliminal control goal priming and subliminal neutral goal priming condition. The order of the two sessions was counterbalanced between the participants. There were two blocks in each session. At the beginning of each block, instructions were given to make a Go response for male (or female) faces and a No-Go response to female (or male) faces. The sequence of the two blocks was counterbalanced across participants. One session consisted of 84 Go-happy trials and 36 No-Go-angry trials in Block 1, and 84 Go-angry trials and 36 No-Go-happy trials in Block 2. Ten practice trials were conducted before each block. The priming task was presented on a 60-Hz Cathode Ray Tube (CRT) screen. In each trial, a fixation was presented for 500 ms. Then a premask was presented for 100 ms, followed by a 20-ms priming word and a 100-ms postmask. Then, an angry or a happy facial picture was presented for 500 ms until the participant made a response (Figure 1). The participants were asked to press the “P” key with their right pointer fingers in response to the female (male) faces and to make no response to the male (female) faces in the Go female/No-Go male (Go male/No-Go female) block. A blank screen was presented for 800–1,200 ms at the end of the trial. Three minutes’ rest was given between blocks and between sessions.

After the main experiment, participants were asked to perform a forced-choice test to examine whether the priming words were perceived consciously. First, a fixation was presented for 500 ms. Then, as in the main experiment, a 20-ms priming word was presented after a 100-ms premask and before a 100-ms postmask. Next, a pair of prime and foil words was presented on the left or the right of the central fixation on the screen. Participants were asked to choose words that had been shown in the trial. The words were presented until a choice was made. Afterward, a blank screen was presented for 1,000 ms before the next trial. Participants were informed that only response accuracy, not response speed, was important.

## Results

### Manipulation Check

A manipulation check for the priming showed participants were unable to consciously categorize the words used for priming in the forced-choice prime categorization task. There was no correlation between the size of the priming effect and the prime visibility in both the subliminal control goal priming (*r* = 0.02, *p* > 0.05) and neutral goal priming conditions (*r* = −0.13, *p* > 0.05), indicating that the control/neutral goals were indeed presented under the perception threshold. At the end of the experiment, all participants reported no awareness of the priming words during the formal experiment.

The mean percentages and standard deviations of hits and false alarms as well as mean RTs to Go stimuli are presented in Table 1.

**TABLE 1:** Behavioral performance in the Go/No-Go task.

	MDD group (*n* = 31)	Control group (*n* = 28)
**Hits (%)**		
Go angry (SD)	86.9 (1.3)	87.6 (1.3)
Go happy (SD)	92.4 (1.0)	93.9 (1.0)
**False alarms (%)**		
No-Go angry (SD)	21.1 (2.4)	16.5 (2.5)
No-Go happy (SD)	15.6 (2.5)	9.7 (2.7)
**Reaction times (ms)**		
Go angry (SD)	466.3 (11.3)	508.9 (11.9)
Go happy (SD)	455.3 (11.3)	491.4 (11.9)

### Main Results

**FIGURE 2:**
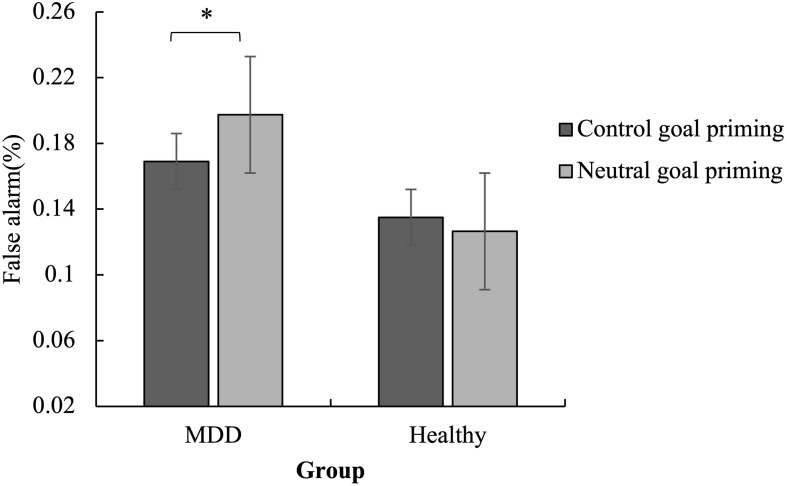
Mean false alarms for the No-Go trials as a function of group and priming. The false alarm was lower for MDD patients in the subliminal control goal priming condition than in the subliminal neutral goal priming condition. No significant difference was found for healthy controls. ^∗^*p* < 0.05.

**FIGURE 3:**
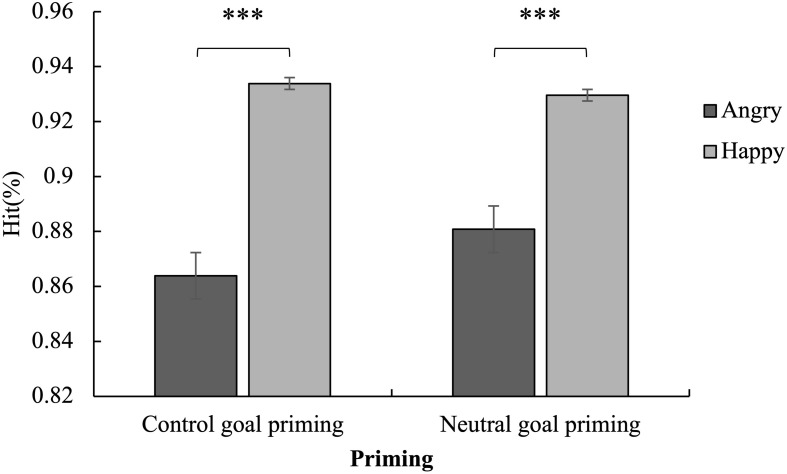
Mean hits for the Go trials as a function of emotion and priming. The hits for happy stimuli were higher than for angry stimuli in both the subliminal control and neutral goal priming conditions. ^∗∗∗^*p* < 0.001.

**FIGURE 4:**
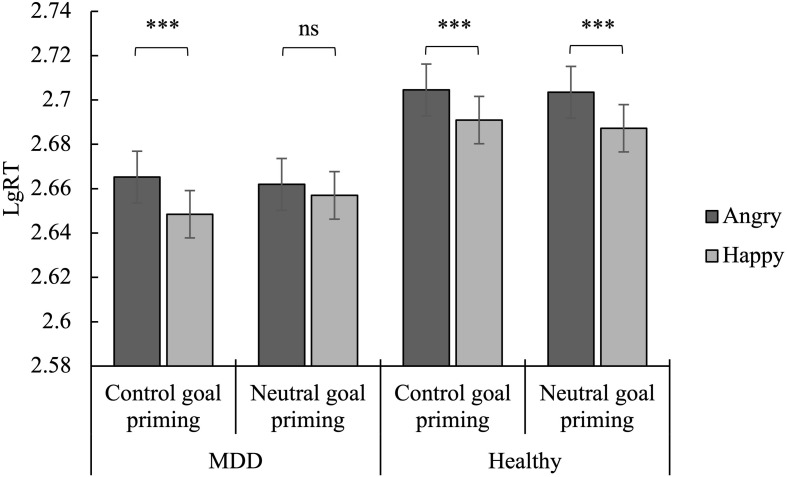
Mean reaction time of Go trials as a function of emotion, priming, and group. ^∗∗∗^*p* < 0.001; ns, no significance.

A three-factor mixed analysis of variance of group × priming × emotion was performed on the false alarms (Figure 2), hits (Figure 3), and reaction time (Figure 4).

#### False Alarm Rates in No-Go Trials

The false alarms showed that the main effect of emotion was significant [*F*(1,57) = 49.20, *p* < 0.001, η*_*p*_*^2^ = 0.46] as the false alarms for angry stimuli were higher than for happy stimuli. The interaction between priming and group was significant [*F*(1,57) = 4.05, *p* < 0.05, η*_*p*_*^2^ = 0.07].

The simple effect analysis revealed that the false alarm was lower for MDD patients in the subliminal control priming condition than in the subliminal neutral goal priming condition (*p* < 0.05). No significant difference was found for the healthy controls (*p* > 0.05). Meanwhile, in the neutral goal priming condition, MDD patients had marginally significantly higher false alarm rates than healthy controls (*p* < 0.10) (Figure 2).

#### Hit Rates in Go Trials

The hits showed that the main effect of the emotion was significant [*F*(1,57) = 119.78, *p* < 0.001, η*_*p*_*^2^ = 0.68] as the hits for happy stimuli were higher than those for angry stimuli. The interaction of emotion and priming was significant [*F*(1,57) = 8.09, *p* < 0.01, η*_*p*_*^2^ = 0.12].

A simple effect analysis of the interaction revealed that the hits for happy stimuli were higher than for angry stimuli in both the subliminal control and subliminal neutral priming conditions (*p* < 0.001) (Figure 3). It is worth noting that the difference between the hits for happy and angry stimuli in the subliminal control priming condition was significantly more than in the subliminal neutral goal priming condition. Additionally, the hits for angry faces in the subliminal control priming condition were lower than in the neutral condition (*p* > 0.05). The hits of responses to happy faces in the subliminal control priming condition were higher than in the neutral condition (*p* > 0.05). However, both results were not significant.

#### Reaction Time

The main effect of emotion was found to be significant [*F*(1,57) = 68.89, *p* < 0.001, η*_*p*_*^2^ = 0.55], indicating a faster response to happy stimuli than to angry stimuli. The main effect of group was also significant [*F*(1,57) = 6.61, *p* < 0.05, η*_*p*_*^2^ = 0.10], indicating that MDD patients reacted faster than healthy controls. Finally, the priming × emotion × group interaction was significant [*F*(1,57) = 6.06, *p* < 0.05, η*_*p*_*^2^ = 0.10].

The simple effect analysis revealed that, for MDD patients, the reaction time to happy faces was significantly shorter than angry faces in the subliminal control goal priming condition (*p* < 0.001). This was not significant in the subliminal neutral goal priming condition (*p* > 0.05). For the healthy controls, the reaction time to the happy faces and angry faces was significant in both the subliminal control and neutral goal priming conditions (*p* < 0.001) (Figure 4).

## Discussion

By employing a subliminal priming technique and an emotional Go/No-Go task, we examined the effect of AER on response inhibition to angry and happy faces among healthy controls and patients with MDD. The results partly supported the hypotheses of the present study. First, in No-Go trials, MDD patients showed lower false alarm rates in the subliminal control goal priming condition than in the subliminal neutral priming condition. No such pattern was found for healthy participants. Meanwhile, happy faces had lower false alarm rates than angry faces. Second, the results of hit rates in Go trials showed that happy faces had greater hit rates than angry faces. Finally, reaction time in Go trials showed the main effects of group and emotion and found a three-way interaction between group × priming × emotion. Simple effect analysis revealed that MDD patients’ reaction time to happy faces was shorter than to angry faces in subliminal control goal priming but not subliminal neutral priming. Healthy controls showed faster responses to happy faces than angry faces in both priming conditions. The findings provided evidence that the subliminal priming of emotion control goals could modulate the response inhibition to emotional faces in MDD patients and revealed the difference in behavioral patterns for AER between MDD patients and healthy controls.

The present study revealed that MDD patients’ false alarm rates were decreased in subliminal control goal priming compared to subliminal neutral priming. Consistently, previous studies indicated that patients with MDD had weaker inhibition during No-Go responses (De Lissnyder et al., 2010; Schlosser et al., 2013; Everaert et al., 2017). Research has also found that higher depression scores were related to a greater tendency to make a Go response in No-Go trials for depressed participants (Kaiser et al., 2003; Ladouceur et al., 2006). Schlosser et al. (2013) also evidenced that MDD patients showed higher false alarm rates during No-Go trials than healthy controls in a Go/No-Go task with fearful, happy, and neutral stimuli. Additionally, compared with subliminal neutral priming, MDD patients’ false alarm rate was decreased in subliminal control goal priming in the present study. A possible explanation could be that the subliminally priming word was closely related to regulation and a control scheme. When participants were primed by regulation-related words and then exposed to an emotional situation, the goal of emotion regulation would be activated automatically and prompt the regulation of emotion. Previous findings have also evidenced that arousal of anger indexed by skin conductance level would be decreased by subliminal control goal priming (Zhang et al., 2017). Furthermore, an emotional Go/No-Go task may reflect the early stage of response inhibition and be related to restraining an impulsive act before it is initiated (Allen and Hooley, 2019), while stop signal tasks may reflect the late stage of response inhibition and reveal the processing of suppression of actions (Allen and Hooley, 2019). Therefore, the present study suggests that subliminal control goal priming would facilitate the inhibition performance in the early stage of response inhibition. Whether subliminal goal priming would affect the late stage of response inhibition of emotions in MDD patients should be evaluated in future studies.

The results also showed that MDD patients reacted faster to happy faces than angry faces in the subliminal control goal priming condition, but not in the subliminal neutral priming condition. Depressed patients tend to have reduced attention to positive stimuli and difficulties with the regulation of positive emotions (Vanderlind et al., 2020). As evidenced by data on eye gaze, depressed participants may lack attentional bias to positive information when asked to watch positive, dysphoric, aversive, and neutral words (Ellis et al., 2011). In the present study, MDD patients were subliminally primed by regulation-related words. When participants made Go or No-Go responses to the sex of faces, emotional information of faces would be interrupters and require regulation. The subliminally primed control goals would prompt automatic regulation processing and decreased emotional arousal to faces. The decreased arousal to happy faces would trigger MDD patient’s avoidance of happy faces and prompt the speed of response to happy faces. However, this hypothesis needs more evidence. Combined with lower false alarm rates of happy No-Go trials for MDD patients in subliminal control goal priming than neutral conditions, the results of the present study may indicate that priming control goals subliminally facilitated MDD’s performance to happy faces.

Interestingly, we found that compared with angry faces, happy faces had higher hit rates, faster responses in Go trials, and lower false alarms in No-Go trials than angry faces. This happy face advantage is consistent with previous studies that found positive facial expressions were recognized more quickly than negative emotions (Albert et al., 2010; Schlosser et al., 2013; Grunewald et al., 2015). For example, Erickson et al. (2005) used an emotional Go/No-Go task and found that participants had shorter latency and higher accuracy in response to happy words than sad words. Similarly, Schulz et al. (2007) found a happy face advantage for an emotional Go/No-Go task by showing faster responses to and higher commission errors with happy faces than sad faces. The findings of the present study suggested that the happy faces advantage may occur even when happy faces were compared with angry faces; this is also related to approach motivation.

However, the results did not find a significant three-way interaction of group × priming × emotion on false alarm rate. We conducted paired-samples *t*-tests to the false alarm rates of angry and happy faces in the subliminal neutral priming condition for two groups separately. Results showed a higher false alarm rate to angry faces relative to happy faces for MDD patients [*t*(30) = 3.72, *p* < 0.01], as well as for healthy controls [*t*(27) = 4.55, *p* < 0.001]. One possible explanation was that anger was related to the approach motivational system (Carver and Harmon-Jones, 2009). Previous empirical studies have shown that angry faces could induce approach behaviors of the participants (Adams et al., 2006; Wilkowski and Meier, 2010). In the present study, the approach motivation in angry trials would lead a response tendency to angry face when an inhibition response was required for both of the two groups. In addition, the present study showed a marginal significant interaction effect of priming and group on the false alarm rate (*p* < 0.10). In the neutral goal priming condition, MDD patients had significantly higher false alarm rate than healthy controls in the present study. This finding suggested that MDD patients had an impairment in emotional response inhibition at the subliminal neutral priming condition compared to the healthy controls. In order to examine whether MDD had abnormalities in the response inhibition of negative affective stimuli than healthy controls, future studies should use sad faces as the stimuli in the task.

It may be questioned whether goals or general concepts were primed by regulation-related verbs in the priming task. The priming materials were referred from Mauss et al. (2007), who evidenced that manipulating emotion regulation-related verbs would activate regulatory goals implicitly. In our procedure, emotion regulation verbs were first primed subliminally, and then emotional facial pictures were presented. Participants were asked to make a Go or a No-Go response according to the sex of the facial picture. The emotions of facial pictures were interrupters, which participants needed to control and regulate when they were finishing the task. Perceptions of emotion and controls of emotional interruption became conflicted processes. Under this situation, the activation of control norms would prompt a process of emotion regulation. One of the principles of goal activation is that goal priming effects should involve inhibition of conflicting goals (Förster et al., 2007). The effect of subliminal control goal priming in MDD patients suggests that control goal-related words activated regulation goals in the present study.

One limitation of the current study is that deliberated emotion regulation or habituated regulation strategies may have been used when participants were finishing the experimental task. The results would be affected by these extraneous variables. In future studies, deliberated emotion regulation used in the experiment should be checked after the experiment by using a self-reported scale. Conversely, individual differences in habituated regulation strategies, which would automatically affect the control goal priming and the emotional Go/No-Go responses, should be measured and used as covariables in the analysis. The Emotion Regulation Implicit Association Test (ER-IAT) has been used in several studies to measure automatic emotion control or expression tendency. Future studies should thus examine the effects of individual differences in ER-IAT and subliminal goal priming on emotional response inhibition for patients with depression.

This study explored the influence of AER on emotional response inhibition among those suffering from MDD and their healthy controls by using subliminal priming techniques and an emotional Go/No-Go task. The results showed that MDD patients’ false alarm rate decreased in subliminal control goal priming compared to subliminal neutral priming. Compared to angry faces, the happy face advantage in reaction time was also found in the subliminal control goal priming condition for MDD patients, but not in the subliminal neutral priming condition. These results may indicate that subliminal goal priming in each emotional Go/No-Go trial enhances the response inhibition to negative emotion stimuli for MDD patients. Furthermore, the findings suggest that goal priming would be a key point to trigger AER, thus providing further evidence for the automotive model (Bargh et al., 2001, 2012; Bargh and Williams, 2007).

## Data Availability Statement

The datasets generated for this study are available on request to the corresponding author.

## Ethics Statement

The studies involving human participants were reviewed and approved by the Ethical Committee of Department of Psychology of Renmin University of China. The patients/participants provided their written informed consent to participate in this study.

## Author Contributions

MZ, SW, and JZ mainly contributed to manuscript writing. MZ, JZ, YZ, and SZ mainly contributed to the research design and data collection. MZ, SW, CJ, YC, YW, YZ, and SZ assisted to recruit the participants and complete the experiment. JZ contributed knowledge to the logic of manuscript. NC contributed her intelligence and experience to the language revision of the manuscript. Finally, JZ and CJ were in charge of and supervised the whole process of research. All authors contributed to the article and approved the submitted version.

## Conflict of Interest

The authors declare that the research was conducted in the absence of any commercial or financial relationships that could be construed as a potential conflict of interest.
